# Homeostatic Tissue Responses in Skin Biopsies from NOMID Patients with Constitutive Overproduction of IL-1β

**DOI:** 10.1371/journal.pone.0049408

**Published:** 2012-11-30

**Authors:** Pamela Aubert, Mayte Suárez-Fariñas, Hiroshi Mitsui, Leanne M. Johnson-Huang, Jamie Lynn Harden, Katherine C. Pierson, Joseph G. Dolan, Inna Novitskaya, Israel Coats, Jacob Estes, Edward W. Cowen, Nicole Plass, Chyi-Chia Richard Lee, Hong-Wei Sun, Michelle A. Lowes, Raphaela Goldbach-Mansky

**Affiliations:** 1 Translational Autoinflammatory Disease Section NIAMS/NIH, Bethesda, Maryland, United States of America; 2 Laboratory for Investigative Dermatology, The Rockefeller University, New York, New York, United States of America; 3 Center for Clinical and Translational Science, The Rockefeller University, New York, New York, United States of America; 4 SAIC, Frederick, Maryland, United States of America; 5 Dermatology, NCI/NIH, Bethesda, Maryland, United States of America; 6 Biodata Mining and Discovery Section, NIAMS/NIH, Bethesda, Maryland, United States of America; 7 Laboratory of Pathology, NCI/NIH, Bethesda, Maryland, United States of America; University of Thessaly, Greece

## Abstract

The autoinflammatory disorder, Neonatal-onset Multisystem Inflammatory Disease (NOMID) is the most severe phenotype of disorders caused by mutations in *CIAS1* that result in increased production and secretion of active IL-1β. NOMID patients present with systemic and organ-specific inflammation of the skin, central nervous system and bone, and respond dramatically to treatment with IL-1 blocking agents. We compared the cellular infiltrates and transcriptome of skin biopsies from patients with NOMID (n = 14) before treatment (lesional (LS) and non-lesional (pre-NL) skin) and after treatment (post-NL) with the IL-1 blocker anakinra (recombinant IL-1 receptor antagonist, Kineret®, Swedish Orphan Biovitrum AB, SOBI), to normal skin (n = 5) to assess tissue responses in the context of untreated and treated disease. Abundant neutrophils distinguish LS skin from pre-NL and post-NL skin. CD11c^+^ dermal dendritic cells and CD163^+^ macrophages expressed activated caspase-1 and are a likely source of cutaneous IL-1 production. Treatment with anakinra led to the disappearance of neutrophils, but CD3^+^ T cells and HLA-DR^+^ cells remained elevated. Among the upregulated genes IL-6, IL-8, TNF, IL-17A, CCL20, and the neutrophil defensins DEFA1 and DEFA3 were differentially regulated in LS tissues (compared to normal skin). Important significantly downregulated pathways in LS skin included IL-1R/TLR signaling, type I and II cytokine receptor signaling, mitochondrial dysfunction, and antigen presentation. The differential expression and regulation of microRNAs and pathways involved in post-transcriptional modification were suggestive of epigenetic modification in the chronically inflamed tissue. Overall, the dysregulated genes and pathways suggest extensive “adaptive” mechanisms to control inflammation and maintain tissue homeostasis, likely triggered by chronic IL-1 release in the skin of patients with NOMID.

## Introduction

“Autoinflammatory” diseases have recently been categorized as a group of disorders with primary excessive activation of innate immune responses [1]. Included in this group are the cryopyrin-associated periodic syndromes (CAPS) that are caused by autosomal dominant mutations in the *cold-induced autoinflammatory syndrome 1 (CIAS1)* gene, also called *NLRP3* or *NALP3* (NACHT domain-, leucine-rich repeat-, and PYD-containing protein 3). The encoded protein, cryopyrin, is a component of the NLRP3 inflammasome, a platform that activates caspase-1 to effectively process pro-IL-1β into its bioactive form. Mutations in *NLRP3* lead to autoactivation of the inflammasome and over-secretion of IL-1β. Between 30–40% of clinically diagnosed patients with NOMID have no detectable mutation in *NLRP3* by Sanger sequencing and genetic mosaicism is currently implicated [2]. However, the “mutation negative” patients' phenotype and response to IL-1 blockade does not differ from *NLRP3-*mutation positive patients [3], [4].

CAPS encompasses syndromes of varying severity, including the mostly familial forms, Familial Cold Autoinflammatory Syndrome (FCAS) and Muckle Wells Syndrome (MWS), and the sporadic, most severe clinical phenotype, Neonatal-onset Multisystem Inflammatory Disease (NOMID). In NOMID, the skin, joints, and central nervous system are affected, manifesting as rashes, clinically resembling urticaria, arthropathy, bony overgrowth, hearing and vision loss, chronic aseptic meningitis, hydrocephalus and developmental delay [5], [6]. Treatment of NOMID patients is by lifelong injection with recombinant IL-1 receptor antagonist (recombinant IL-1Ra, anakinra, Kineret®, Swedish Orphan Biovitrum AB, SOBI) [3]. This promises to help prevent long-term sequelae of inflammation, and studies with additional IL-1-blocking agents are ongoing [7].

Up-regulation of IL-1β production is seen in monocytes from all patients with CAPS [6], [8] but cryopyrin is also expressed in keratinocytes, macrophages, chondrocytes and resident mast cells [9]. However, the functional contribution of IL-1β production from these cells to the inflammatory tissue phenotype remains unknown. The urticarial-like skin lesions in NOMID can be distinguished histologically from allergic urticaria by a dense dermal neutrophilic infiltrate [3], rather than an eosinophilic infiltrate. In untreated patients, the severity of the urticaria-like lesions varies, and the rash can even disappear completely without treatment. The spontaneous resolution of lesions in untreated patients raises the possibility of tissue specific counter-regulatory pathways that dampen the inflammatory effect of chronic IL-1 release in these patients.

In this paper, tissue samples from the skin of patients with NOMID were studied by immunohistochemistry and by gene expression array. Pre-treatment skin, both lesional (LS) biopsies from erythematous involved skin and non-lesional (pre-NL) biopsies from clinically normal appearing skin, were compared with post treatment skin biopsies where the urticaria-like lesions had resolved (post-NL), and with normal skin from healthy donors. Neutrophil elastase-positive cells (neutrophils) in the dermis, and alpha-defensin genes (DEFA1 and DEFA3) on microarray distinguish LS from pre-NL tissue. A surprisingly high number of over two thirds of differentially regulated genes were downregulated in LS tissue compared to normal skin. Pathway analyses suggest extensive regulation of adaptive pathways including the downregulation of proinflammatory cytokine pathways and pathways regulating epigenetic modifications in an attempt to prevent tissue damage and regain tissue homeostasis.

## Results

### NOMID LS skin was characterized by a dermal neutrophilic infiltrate and activated caspase-1 in myeloid cells

**Figure 1:**
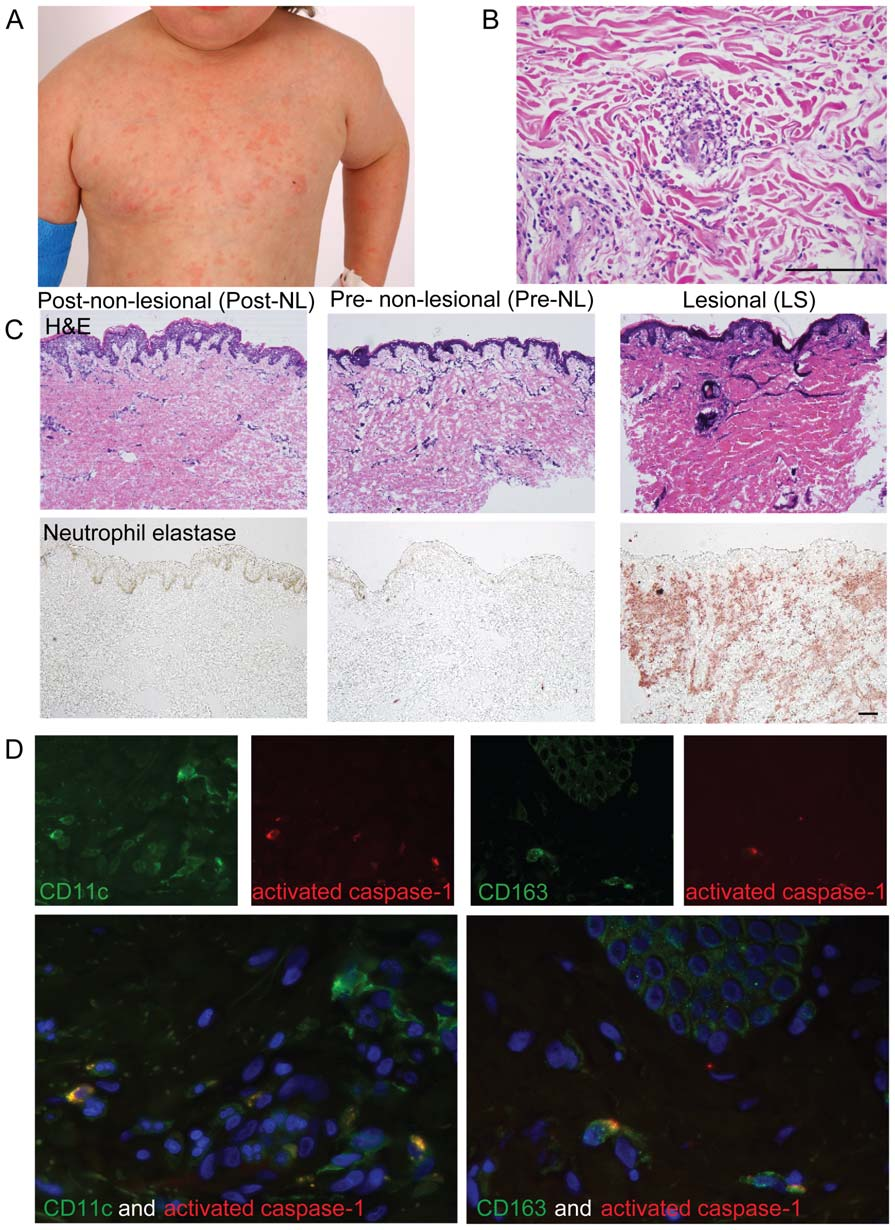
Clinical and histological features of patients with NOMID. **A.** Appearance of the classical urticaria-like rash of NOMID, with scattered erythematous papules, perhaps better termed “IL-1-mediated neutrophilic dermatosis (IMEND)”. **B.** Representative haematoxylin and eosin (H&E) staining of a lesional skin biopsy showing prominent dermal neutrophilic urticaria. **C.** Comparison of representative post-NL, pre-NL, and LS biopsies by H&E (upper panel) and neutrophil elastase (lower panel) immunohistochemistry, showing the presence of neutrophils only in LS biopsies, with normal overlying epidermis. **D, E.** Immunoflourescence of LS biopsies showing activated caspase-1 (red) in both CD11c^+^ dendritic cells and CD163^+^ macrophages (green: double positive cells are yellow). Size bar is 100 µm.

The morphology of the lesions was of erythematous papules and plaques, which clustered locally or developed into a generalized eruption, clinically resembling lesions of allergic urticaria (Fig. 1A). As previously described, the typical lymphocytic or eosinophilic inflammatory infiltrate of classic urticaria was absent in the NOMID skin biopsies, and a superficial and deep perivascular and interstitial infiltrate predominantly composed of neutrophils was present (Fig. 1B) [3], [10]. Normal skin is shown for comparison (Fig. S1A). The epidermis was histologically unremarkable. Neutrophils and neutrophil elastase staining was only seen in LS tissue, and was absent in post-NL and pre-NL samples (Fig. 1C). Some of the neutrophils demonstrated string-like, extracellular elastase and DAPI immunofluorescent co-staining in the dermis that could be consistent with neutrophil extracellular traps (NETs, Fig. S2). In contrast with psoriasis, in which NET-like structures localized to the epidermis, this staining in NOMID was localized to the dermis.

As *NLRP3* mutations lead to caspase-1 activation and subsequent IL-1β release [11]–[13], we found activated cleaved caspase-1 positive cells (red) among dermal CD11c^+^ dendritic cells and dermal CD163^+^ macrophages (green); co-expression of caspase-1 and the cell marker appear as yellow cells (Fig. 1D left and right, respectively). There was no caspase-1 staining in the epidermis, suggesting that dermal myeloid cells and macrophages are a likely source for production of bioactive IL-1β in NOMID skin. IL-1Ra and also IL-36Ra expression in any of the NOMID inflammatory tissue states did not differ markedly from normal skin (Fig. S1B and Fig. S1C).

### Myeloid derived cells and T cells were increased in pre-NL and LS NOMID skin

**Figure 2:**
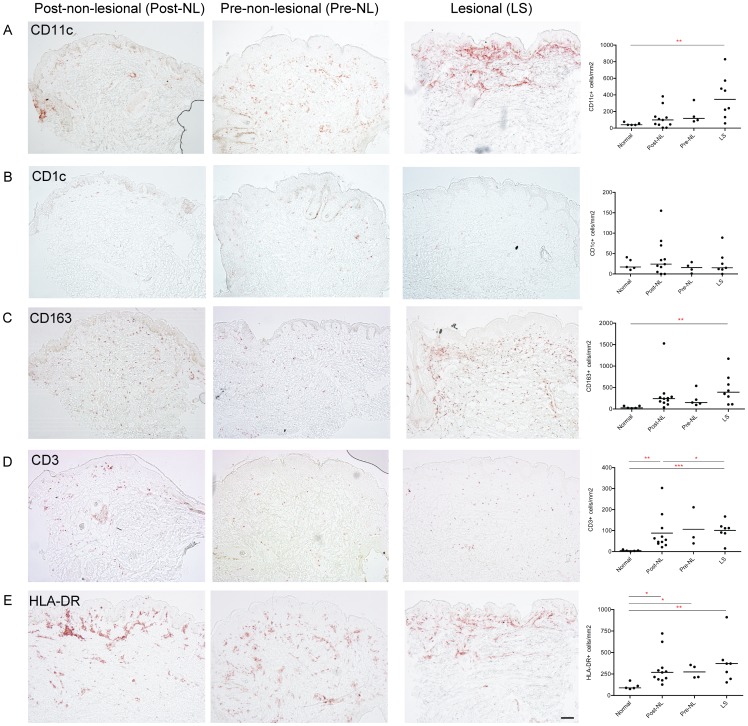
Myeloid cells and T cells were abundant in pre-NL and LS NOMID skin. Representative immunohistochemistry for post-NL, pre-NL, and LS skin biopsies, and cell counts/mm^2^ dermis. **A.** There were abundant CD11c^+^ inflammatory myeloid dendritic cells in all NOMID tissue groups. **B.** There were no differences in the CD1c^+^ resident dendritic cells across the groups. **C, D, E.** There were abundant CD163^+^ macrophages, CD3^+^ T cells, and HLA-DR^+^ activated cells in NOMID tissues. *p<0.05, **p<0.01, ***p<0.001. Size bar is 100 µm.

We next analyzed the number of resident and inflammatory dendritic cells (Fig. 2), which we have previously defined as CD11c^+^CD1c^+^, and CD11c^+^CD1c^−^, respectively [14]. LS skin had significantly increased numbers of CD11c^+^ myeloid dendritic cells compared to normal skin (*p* = 0.003), and the distribution was predominantly dermal [14] (Fig. 2A). Although there was a trend towards more CD11c^+^ dendritic cells in LS compared to pre-NL and post-NL skin, these comparisons were not statistically different. As the number of “resident” CD1c^+^ myeloid dendritic cells was similar in all groups studied (Fig. 2B), this suggests that the CD11c^+^ “inflammatory” DCs were elevated in LS tissues. Dermal CD163^+^ macrophages were present in all NOMID groups, with statistically increased CD163^+^ cells in LS compared to normal skin (*p* = 0.012) (Fig. 2C).

CD3^+^ T cells were similarly increased in the dermis of pre-NL and LS NOMID skin as well as post-NL skin, compared to normal healthy controls, (*p* = 0.0009 for LS versus normal and *p* = 0.014 for post-NL versus normal skin) (Fig. 2D). CD3^+^ T cells decreased in post-NL skin with treatment and were significantly lower compared to LS skin (*p* = 0.03). HLA-DR^+^ cells (representing antigen presenting cells and activated T cells) were also increased throughout all NOMID samples compared to normal skin, with significant increases in HLA-DR^+^ cells between LS and normal skin (*p* = 0.005), pre-NL and normal skin (*p* = 0.05), and post-NL and normal skin (*p* = 0.04) (Fig. 2E).

### Gene expression shows a “severity gradient” of skin abnormalities from normal skin to post-NL, pre-NL and LS NOMID skin

**Figure 3:**
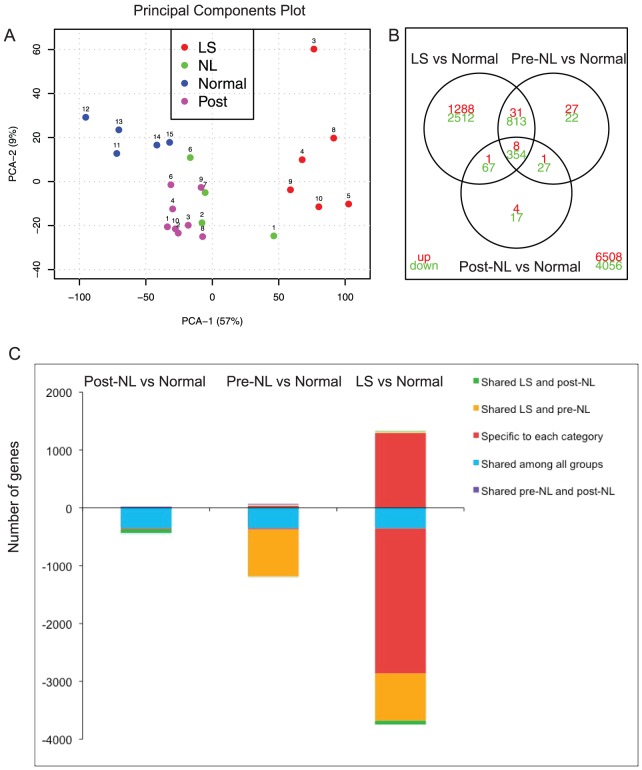
Gene expression of LS, pre-NL, post-NL and normal skin. **A.** Principal Component Analysis (PCA) of samples used in this study, with PCA1 and PCA2 explaining together 66% of the variance. Number identifies each patient. **B** Venn diagram showing number of *uniquely* upregulated (red) or downregulated (green) genes comparing LS, pre-NL and post-NL to normal tissues (*total* probe-sets in lower right corner). **C.** Bar diagram indicating shared differentially expressed genes (DEGs) between tissue states in the same color. Most DEGs in post-NL tissue were also differentially regulated in pre-NL and LS tissue when compared with normal skin, and these are depicted as a blue block. Additional DEGs shared between LS and pre-NL (orange), shared between LS and post-NL (green), and shared between pre-NL and post-NL (purple) are shown. A large number of specifically regulated DEGs were only found in LS (red). Hence, there was a “crescendo effect” with additional numbers of up- and down-regulated DEGs recruited as the tissue became more diseased and inflamed.

Principal Component (PC)1 versus PC2 analysis of these specimens shows the clustering of individual samples into groups (Fig. 3A). Post-NL skin samples were clustered “between” normal and pre-NL skin. LS skin clustered away from these other groups. The immunohistochemistry results as well as the clustering results indicate a gradient of tissue involvement ranging from normal to post-NL to pre-NL to LS as the most distant from normal skin.

Differentially expressed genes (DEGs) in each diseased tissue group compared to normal were determined (>2 fold change [FCH], false discovery rate [FDR] <0.05; Table S1). The number of DEGs is summarized in the Venn diagram (Fig. 3B) and the expression values are represented in a heatmap (Fig. S3). It was striking that the majority of DEGs were downregulated in the diseased states, especially in LS. While 14, 67 and 1328 unique genes were upregulated in post, pre-NL and LS respectively, there were 465, 1216 and 3746 significantly downregulated genes. Fig. 3C shows the number of shared DEGs among tissue states, in the same color. Most DEGs in post-NL tissue were also differentially regulated in pre-NL and LS tissue when compared with normal, and these are depicted as a blue block. Additional DEGs were shared between LS and pre-NL (orange), between LS and post-NL (green), and between pre-NL and post-NL (purple). Furthermore, a large number of DEGs were only found in LS (red). Hence, there was a “crescendo effect” with the recruitment of additional up and downregulated DEGs, as the tissue became more diseased and inflamed.

### Inflammasome components, IL-1 and IL-1 receptor family members, and other inflammatory cytokines and chemokines were differentially regulated in the various tissue states compared to healthy tissues

**Figure 4:**
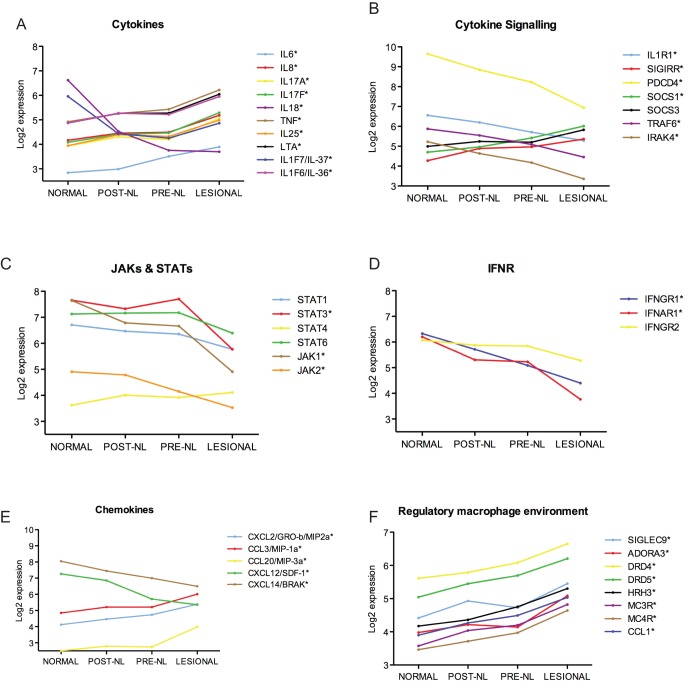
Level of expression of selected genes in normal, post-NL, pre-NL and LS NOMID skin. Gene expression for **A.** cytokines, **B.** cytokine signaling, **C.** JAKs and STATs, **D.** interferon receptors (IFNR), **E.** chemokines and, **F.** receptors that respond to mediators that induce regulatory macrophages. Asterisk indicates gene is significantly differentially regulated in LS compared to normal skin (p<0.0006) (gene expression values from Table S2, DEGs and *p* values from Table S1).

Estimators of the mean gene expression (in log_2_-scale) were obtained for LS, pre-NL, post-NL and normal skin (Table S2) and a selection of them is plotted in Fig. 4. The expression of a number of proinflammatory cytokines was significantly upregulated in the LS state compared to normal skin (Fig. 4A). IL-6 was upregulated in both pre-treatment tissue states, LS (*p* = 7.72×10^−6^) and pre-NL (p = 0.0012). Other pro-inflammatory cytokines including IL-17A (p = 1.32×10^−5^), IL-17F (p = 3.86×10^−8^), IL-25 (also IL-17E, p = 7.66×10^−9^), TNF (p = 2.47×10^−8^), IL-8 (p = 1.59×10^−5^), Lymphotoxin-α (p = 1.87×10^−7^), and IL-1F6/IL-36α (p = 3.02×10^−6^) were significantly upregulated only in LS tissues. In contrast, two other interleukins that require enzymatic cleavage for activation, IL-18 and IL1F7/IL-37 [15]–[17] were significantly downregulated in different tissue states compared to normal skin (for IL-18: p = 1.63×10^−6^ and for IL1F7/IL-37 two probesets: p = 9.28×10^−5^ and 2.59×10^−8^), with the most pronounced downregulation for both in LS tissues.

Transcripts for IL-1 could not reliably be detected, as they were filtered out of the analysis due to low expression, as previously described for many cytokine transcripts [18]. However the IL-1 signaling pathways were examined by a candidate gene approach and ingenuity pathway analysis (IPA) (Fig. 4B and Fig. S4). In LS tissues, the gene expression for the IL-1 Type1 receptor was significantly downregulated, which is in contrast to upregulation for the anti-inflammatory receptor SIGIRR, known to suppress the IL-1 receptor pathway [19]. The expression of key signaling molecules of the IL-1R1/TLR was also downregulated including IRAK-4, TRAF6 and PDCD4. In contrast, gene expression for the negative regulators, suppressor of cytokine signaling (SOCS) 1 and also SOCS3 (the latter not statistically significant), were most upregulated in LS tissues. Molecules associated with signaling through Type I and II cytokine receptors such as STAT3 and Jak1 and 2, but not STAT1 and STAT4, were all significantly downregulated in LS tissues (Fig. 4C). Gene expression for IFNγRI and IFNαRI receptor chains were most significantly downregulated in LS tissue (Fig. 4D). IPA analysis confirmed that many genes in the *IL-1* and *JAK/STAT signaling* canonical pathways were significantly downregulated in LS skin (shown in green with upregulated genes in red, Fig. S4).

Expression of chemokines was variable (Fig. 4E). Neutrophil chemokines IL-8 (Fig. 4*A*) and the CXC chemokine CXCL2/GROβ/MIP2 (Fig. 4E) were upregulated in LS tissue [20], [21]. CCL3/MIP1α, a chemokine for inflammatory monocytes that is often upregulated in IL-1-mediated diseases [21], was also increased in LS skin. The CC chemokine CCL20/MIP3α, which can be produced by neutrophils and is a chemotactic factor for CCR6^+^ T cells, B cells, and dendritic cells [22], [23] was also upregulated in LS tissue. In contrast, broad chemokines CXCL14 and CXCL12 [20] were both downregulated in LS skin. Finally, regulatory macrophages may be induced by adenosine, dopamine, histamine, sphingosine-1-phosphate, melanocortin, vasoactive intestinal peptide, adiponectin and Siglec-9 (reviewed in [24]). Gene expression for the receptors for many of these agents were increased in LS skin (Fig. 4F), suggesting an environment in LS skin conducive to induce regulatory macrophages that can limit inflammation.

### Canonical pathways that were differentially regulated in the different NOMID tissue states may be involved in tissue homeostasis

IPA was used to identify pathways that were significantly over-represented by the DEGs in the comparison of interest (Table S3). The pathways represented by the differentially upregulated genes in LS versus normal tissues included *acute phase response signaling*, *amyloid processing*, *IL-6 and IL-17 signaling* and *Wntβ/catenin signaling* pathways. Although these pathways were significantly over-represented by upregulated DEGs in LS tissues, it should be pointed out that in all these pathways a large number of DEGs were individually downregulated, significantly so in the *acute phase response signaling*, *IL-6 signaling* and *IL-17 signaling* pathways. Hence, genes in these pathways were both upregulated and downregulated, suggesting that in the presence of active inflammation there is “an effort” to dampen the impact of inflammation.

The rash severity in NOMID may fluctuate significantly from an involved area to a clinically normal area, before treatment with anakinra or any other therapy including steroids (Fig. S5). These states are represented by LS and pre-NL skin biopsies, and DEGs are presented in Table S1. Only 76 unique genes were up-regulated in LS skin compared to pre-NL skin, yet there were over 20 times more downregulated genes in LS skin compared to pre-NL skin (1632 genes). Key genes that were upregulated in LS skin included two α-defensins (DEFA1 and DEFA3), which are abundant in neutrophils, other proinflammatory cytokines such as IL-6, IL-8, and the anti-apoptotic mediator BCL2A1. We hypothesized that the genes downregulated in LS tissue may provide an understanding of the processes that lead to resolution of the inflammatory skin lesion (conversion from LS to pre-NL skin). The top canonical pathways that were represented by these differentially downregulated genes (Table 3SB) are suggestive of: 1. epigenetic modification, (i.e. *DNA Methylation and Transcriptional Repression Signaling*, *Cleavage and Polyadenylation of Pre-mRNA*, *Protein Ubiquitination Pathway*); 2. modification of mitochondrial function, (i.e. *Oxidative Phosphorylation* and *Mitochondrial Dysfunction* (Fig. S6A); 3. cell transport (i.e. *Clathrin-mediated Endocytosis Signaling*); and 4. a mechanism that serves to maintain intracellular redox homeostasis and limit oxidative damage (i.e. *NRF2-mediated Oxidative Stress Response*). In addition, genes in pathways that are associated with antigen presentation, MHC class I and class II genes including β2 microglobulin for MHC class I, and the invariant chain (Ii) respectively, were significantly downregulated. Components of the constitutive and induced proteasomes which are involved in protein degradation and antigen presentation were also mostly downregulated [25] (Fig. S6B and Fig. S6C).

### Evidence of epigenetic regulation is found in the most inflamed tissue states

Based on the observation that a large number of genes were downregulated in LS skin compared to normal skin and pre-NL skin, we looked for evidence of epigenetic regulation, including histone and DNA modification, presence of microRNAs (mirs) and RNA post-translational modification. Interestingly, the expression levels of the majority of these genes in LS tissue were decreased, including the enzymes associated with histone modification and histone gene expression (Fig. S7A), but a small number of histone gene expression levels were also increased. MicroRNA expression levels also varied across tissue states. While most of the microRNAs were upregulated in the LS tissue, only two microRNAs, mir29c and mir103-2, were significantly downregulated (Fig. S7B), and these have been shown to be involved in tissue fibrosis surrounding a cardiac infarct [26], and in the modulation of IGF-1 mediated stabilization of mRNA [27], respectively. Other mirs that have been reported to be skin specific were upregulated in LS tissues; these include mirs 9-1, 199a-2, 203, and 320a [28]. The *DNA Methylation* and *Polyadenylation of pre-mRNA* pathways were also significant in LS skin (Table S3). Overall, there appears to be complex epigenetic regulation of genes in the skin, suggesting additional levels of genetic control during this inflammatory process. Bcl-3, which controls LPS induced TNF-alpha production [29], IL-10 and IL-1β [30], and HES/HEY-like transcription repressors, two LPS inducible negative regulators that limit inflammation in a gene dependent manner, were also induced in LS tissue.

### Treatment with anakinra leads to clinical resolution of the skin lesion but residual gene expression abnormalities persist

To evaluate the resolution of NOMID after treatment with anankinra, we compared gene expression in post treatment tissue (post-NL) to normal skin (Table S4). There were 14 unique genes that remained upregulated compared to normal tissue and a total of 465 unique genes remained statistically significantly downregulated compared to normal tissues (blue block, Fig. 3C).

## Discussion

NOMID is a model disease to study the effect of chronic IL-1β overproduction in organ tissues. The ‘NOMID rash’ is characteristic and presents as red erythematous macular-papular lesions with a neutrophilic dermal infiltrate, perhaps better named “IL-1-mediated neutrophilic dermatosis (IMEND)”. The severity of disease varies widely in the same individual even without treatment [3], [6]. Using immunohistochemistry and gene profiling we established a “severity gradient” of skin abnormalities from normal skin. Interestingly, clinically “normal” appearing pre treatment (pre-NL) and post treatment skin (post-NL) had significant differences from normal tissues, by immunohistochemistry and by residual gene expression abnormalities. Residual gene expression profile differences have also been seen in psoriatic skin post treatment [31] suggesting that a “sub-clinical” state of inflammation and tissue adaptation can persist.

Immunohistochemical and immunofluorescent staining indicated that a likely source of bioactive IL-1β production in the skin is myeloid cells in LS dermis that contain activated caspase-1, the enzyme that cleaves pro-IL-1β into its active form. The presence of neutrophils is also characteristic of LS skin. Abundant neutrophils in LS are consistent with an increased gene expression for neutrophil defensins α1 and α3 (DEFA1, DEFA3), where they constitute two of the most upregulated genes compared to normal, pre-NL and post-NL skin. Neutrophils are important effector cells in the killing of bacteria and release of cytotoxic granules, but also have the capability to destroy tissue. However, in NOMID, there is preservation of the skin architecture without scarring at any time. Thus, while neutrophils are usually considered to be first-line pro-inflammatory effector cells, it is possible that they may have a dual role in the resolution of inflammation [32]. Recent reports have focused on the anti-inflammatory role of neutrophils such as by releasing neutrophil extracellular traps (NETs) [33], or by their response to IL-10 [34]. The observation that neutrophil laden LS skin can spontaneously revert to a less inflamed tissue state (pre-NL) in untreated NOMID patients could be explained by the short lifespan of neutrophils. In addition, we speculate that neutrophils could instigate negative feed back mechanisms to control inflammatory damage, preserve tissue function, and initiate resolution of inflammation. In fact, the release of α-defensin proteins from neutrophils rendered protection in a murine peritonitis model [35] and blocked phagocytosis [36], indicating another mechanism by which they could control inflammation.

Increased IL-17 mRNA and the role of IL-1 in Th17 cell differentiation [37] have led to consideration of IL-17 as an important downstream mediator in cutaneous inflammation and neutrophil recruitment [38], [39]. However, there are controversial data on the role of IL-17 in CAPS from mouse models, where IL-17 has been described both as either necessary [40], or not required [41], for the recruitment of neutrophils. Our data indicated that despite increased IL-17 mRNA in LS skin compared to normal tissue, there was, a mixed effect on IL-17 signaling (both up and down regulated), and IL-17-responsive keratinocyte genes (as determined by the addition of IL-17 to keratinocytes) were not elevated [38]. The increase in SIGIRR expression seen in inflamed tissue may even suppress Th17 cell proliferation [19]. In addition, in LS skin, there was downregulation of MHC class I and II genes suggesting reduced antigen presentation. Hence, the dysregulation of IL-17-induced genes and antigen-presentation may present a possible protective mechanism to limit the development of autoimmunity in IL-1 mediated auto-inflammation, a feature that is absent in CAPS patients even in patients with longstanding disease.

Two thirds of all DEGs in LS tissue were downregulated, which suggests inflammation-dependent epigenetic modification. Epigenetic effects, which include DNA methylation, histone modification, increased microRNA expression, and chromatin modifications have been found to induce a state of “LPS tolerance” that was observed upon restimulation of mainly monocytes with LPS and led to decreased cytokine responses [19], [42]–[46]. Epigenetic modification in inflamed NOMID skin is supported by the following observations: IPA demonstrated downregulation of genes in the *DNA methylation* and *Transcriptional repression and polyadenylation* pathway; enzymes modifying histones and histone genes were differentially regulated in LS skin; and variations were seen across the tissue states for 36 microRNAs and 46 small nucleolar RNAs (Table S1).

A model of cellular and gene expression/pathway changes that occur in the different tissue states of IL-1 mediated tissue injury summarizes our data (Fig. S8). Increased IL-1 production leads to recruitment of inflammatory cells, particularly neutrophils, into the dermis. Gene expression levels for the IL-1 receptor and their downstream signaling molecules, Type I and II cytokine signaling pathways and IFN receptors, are progressively downregulated as the tissue states become more “inflamed”. In contrast, negative regulators such as the anti-inflammatory receptor SIGIRR, SOCS1, SOCS3, and the IL-10-inducible negative regulator Bcl-3, become progressively upregulated in inflamed tissue, suggesting negative feed back loops to downregulate IL-1 mediated signaling [47]. The increased expression of genes that allow regulatory macrophages to respond to their environment [24], [48] supports the presence of cells whose goal is to induce down-regulatory circuits.

As inflammasome mediated IL-1β production may be involved in causing tissue stress and damage in a number of prevalent human diseases, including Type II diabetes, obesity, and coronary artery disease [49], the understanding of the regulation of the homeostatic pathways that can limit tissue stress and damage and restore tissue homeostasis, may lead to novel treatment strategies that can limit inflammation induced tissue stress and damage, and become a viable option to protect tissue undergoing cytokine induced damage.

## Materials and Methods

### Ethics statement

The study protocol was approved by the National Institute of Arthritis and Musculoskeletal and Skin Diseases/National Institute of Diabetes and Digestive and Kidney Diseases (NIAMS/NIDDK) Institutional Review Board (IRB) in 7/2003. All patients were enrolled in the IRB-approved protocol 03-AR-0298, and written informed consent was obtained from patients and/or their legal guardians. The clinical trial was conducted according to principles expressed in the Declaration of Helsinki (clinicaltrials.gov: NCT00069329).

### Characteristics of patients with NOMID

The demographic and clinical characteristics of patients studied with NOMID (n = 14) are described in Table S5. Age ranged from four years to 28 years. There were six males and eight females, and ten patients were positive for *NLRP3* mutations on Sanger sequencing, while four were negative. Prior to beginning treatment all patients had classical urticaria-like skin lesions. All patients had CNS inflammation including aseptic meningitis, hearing loss, visual impairment, and many had developmental delay [3]. A subset of patients also presented with characteristic bony overgrowth [50]. All patients were treated with anakinra, recombinant IL-1Ra (Kineret®, Swedish Orphan Biovitrum AB, SOBI), at 1.5–2.0 mg/kg/day for 3 months. At the time of repeat biopsy, the rash in all patients had completely resolved. Many patients were on stable or down-tapering doses of oral steroids at the time of biopsy (dosage in Table S5).

### Patients and skin samples

Over the past seven years under this protocol, four mm paired skin biopsies were collected from patients with NOMID (n = 14) at the time of diagnosis (either LS or pre-NL), and after treatment (post-NL) (Table S5). Neonatal normal foreskins were used as normal controls for immunohistochemistry studies. The Rockefeller University IRB approved the collaboration to perform experiments and analysis of de-identified study materials. Archived normal skin samples collected at Rockefeller University were used as controls in the genomic studies. The collection of this clinical material was conducted under a Rockefeller University-IRB approved study, and all patients gave informed consent.

### Immunohistochemistry and Immunofluorescence

Tissue sections were stained in a standard manner as previously published [51], and are outlined further in Methods S1. A list of antibodies is in Table S6.

### RNA preparation, Gene Array, Statistical Analysis

Details are outlined in Methods S1. Briefly, the NuGen system (San Carlos, CA) with Affymetrix Human Gene 1.0 ST arrays (Santa Clara) was used. Microarray analysis was conducted in *R* (www.r-project.org). Probesets with SD>0.4 and at least one sample with expression >3 were included in the analysis. To identify differentially expressed genes (DEG) we used a mixed effect model with fixed effect Group and random intersect for Patient. A steroid dosage was included in the model as a covariate. Genes with FDR<0.05 and FCH>2 were considered DEGs. These lists of DEGs were used for identifying significantly regulated canonical pathways with the Ingenuity Pathways Analysis program (IPA, Ingenuity Systems, www.ingenuity.com). The data discussed in this publication are in compliance with the MIAME guidelines and have been deposited in the National Center for Biotechnology Information's Gene Expression Omnibus and are accessible through Gene Expression Omnibus Series accession number GSE27864.

Further details are provided in the Methods S1 document.

## Supporting Information

Figure S1 **IL-1Ra and IL-36Ra are present in normal skin and in NOMID.** **A.** Normal skin H&E and negative control for immunohistochemistry. **B, C.** IL-1Ra and IL-36Ra expression in normal skin (Normal), post-non-lesional (Post-NL), pre-treatment non-lesional (Pre-NL), and lesional (LS). Size bar is 100 µm.
(TIF)

Figure S2 **Neutrophil Extracellular Trap (NET)-like structures are found in NOMID.** Frozen sections from patients with **A.** psoriasis, or **B.** NOMID were used to stain for neutrophil elastase via immunohistochemistry (left), as well as immunofluorescence of neutrophil elastase (red) and DNA (DAPI) (blue; right, n = 3 for NOMID). NET-like structures can be seen as purple co-localization of neutrophil elastase and DNA. **A.** In psoriasis, these NET-like structures were seen predominantly in the epidermis. **B.** In NOMID, NET-like structures were seen within the dermis. All images are shown at 20×, size bar is 100 µm.
(TIF)

Figure S3 **Heat map for DEGs in study.** Clustering of patients into lesional (LS), pre-treatment non-lesional (NL in figure, pre-NL in text), normal, and post-treatment non-lesional (post, post-NL) groups.
(TIF)

Figure S4 **Ingenuity pathway analysis for** ***IL-1*** **and** ***Cytokine signaling*** **pathways.** DEGs for LS vs N skin were analyzed using Ingenuity Pathways Analysis (IPA) software. These two canonical pathways, **A.** *IL-1* and, **B.** *Cytokine Signaling* pathways were significantly expressed in LS vs N DEGs, with many of the genes in the pathways down-regulated (green) rather than upregulated (red).
(EPS)

Figure S5 **Lesions can improve spontaneously in NOMID.** Example of lesional NOMID skin with spontaneous resolution.
(EPS)

Figure S6 **Ingenuity Pathway Analysis (IPA) results for mitochondrial dysfunction and antigen presentation.** DEGs for LS vs Normal skin were analyzed using Ingenuity program. **A.** This *mitochondrial dysfunction* pathway was significantly expressed in LS versus Normal DEGs, with many of the genes in the pathway downregulated (green) rather than upregulated (red). There were also many downregulated genes in *antigen presentation pathways*. B. MHC Class I pathway and, C. MHC Class II.
(EPS)

Figure S7 **Level of expression of selected histone and microRNA (mir) genes in post-NL, pre-NL and LS NOMID tissue compared to normal skin.** Mean gene expression across patient groups for selected **A.** histones and **B.** mirs. All genes included above were significantly differentially regulated in LS compared to normal skin (gene expression values from Table S2, DEGs from Table S1).
(EPS)

Figure S8 **Model of the progression of inflammation in NOMID.** Pre-non-lesional (pre-NL) to lesional (LS) skin, and improvement with anti-IL-1β treatment. Infiltrating myeloid cells (dendritic cells and macrophages) are activated in NOMID and produce excessive IL-1β, which results in an influx of neutrophils and neutrophil-specific products. In lesional tissue, all cells (macrophages, myeloid DCs, T cells and neutrophils) were significantly increased compared to normal skin. Key pro-inflammatory cytokines were upregulated. Many differentially regulated pathways were evident. These tissue changes suggest adaptive tissue responses geared to downregulate pro-inflammatory stimuli, reduce antigen presentation, prevent the development of autoimmunity, reduce energy production, and to regain and maintain tissue homeostasis.
(EPS)

Methods S1 (DOC)

Table S1 **DEGs for each comparison.**
(XLS)

Table S2 **Average level of gene expression, “mean per group” for each tissue state.**
(XLS)

Table S3 **Ingenuity Pathways Analysis (IPA).**
(PDF)

Table S4 **Residual subclinical disease profile in post-NL skin.**
(XLS)

Table S5 **Clinical details of patients included in the study.**
(PDF)

Table S6 **Antibodies used in this study.**
(PDF)
