# Supplementary figures and images for: Homeostatic Tissue Responses in Skin Biopsies from NOMID Patients with Constitutive Overproduction of IL-1β
Source: PLoS One. 2012 Nov 30;7(11):e49408. doi: 10.1371/journal.pone.0049408 (PMC3511496; doi:10.1371/journal.pone.0049408)

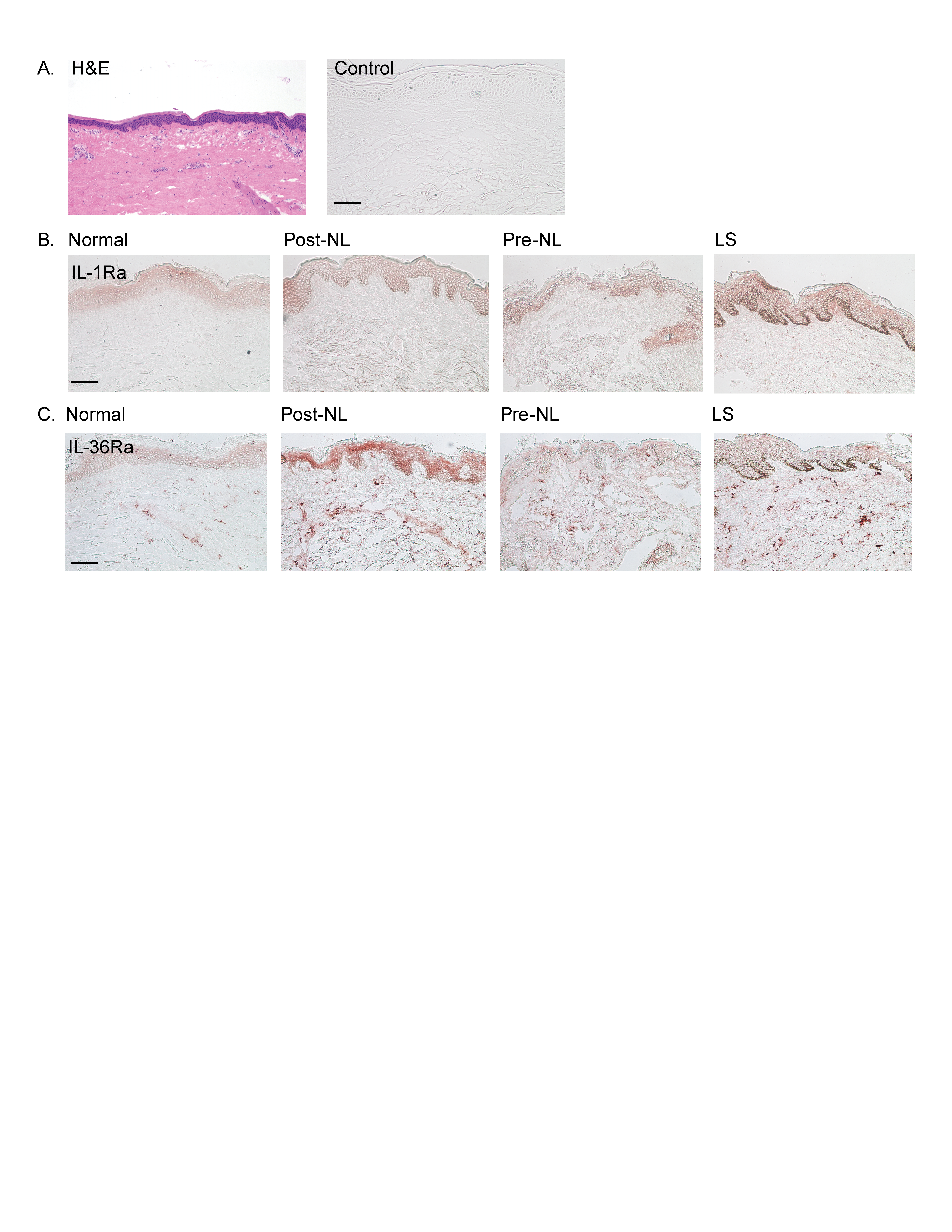

Supplement: Figure S1 — IL-1Ra and IL-36Ra are present in normal skin and in NOMID. A. Normal skin H&E and negative control for immunohistochemistry. B, C. IL-1Ra and IL-36Ra expression in normal skin (Normal), post-non-lesional (Post-NL), pre-treatment non-lesional (Pre-NL), and lesional (LS). Size bar is 100 µm. (TIF) [file pone.0049408.s001.tif]

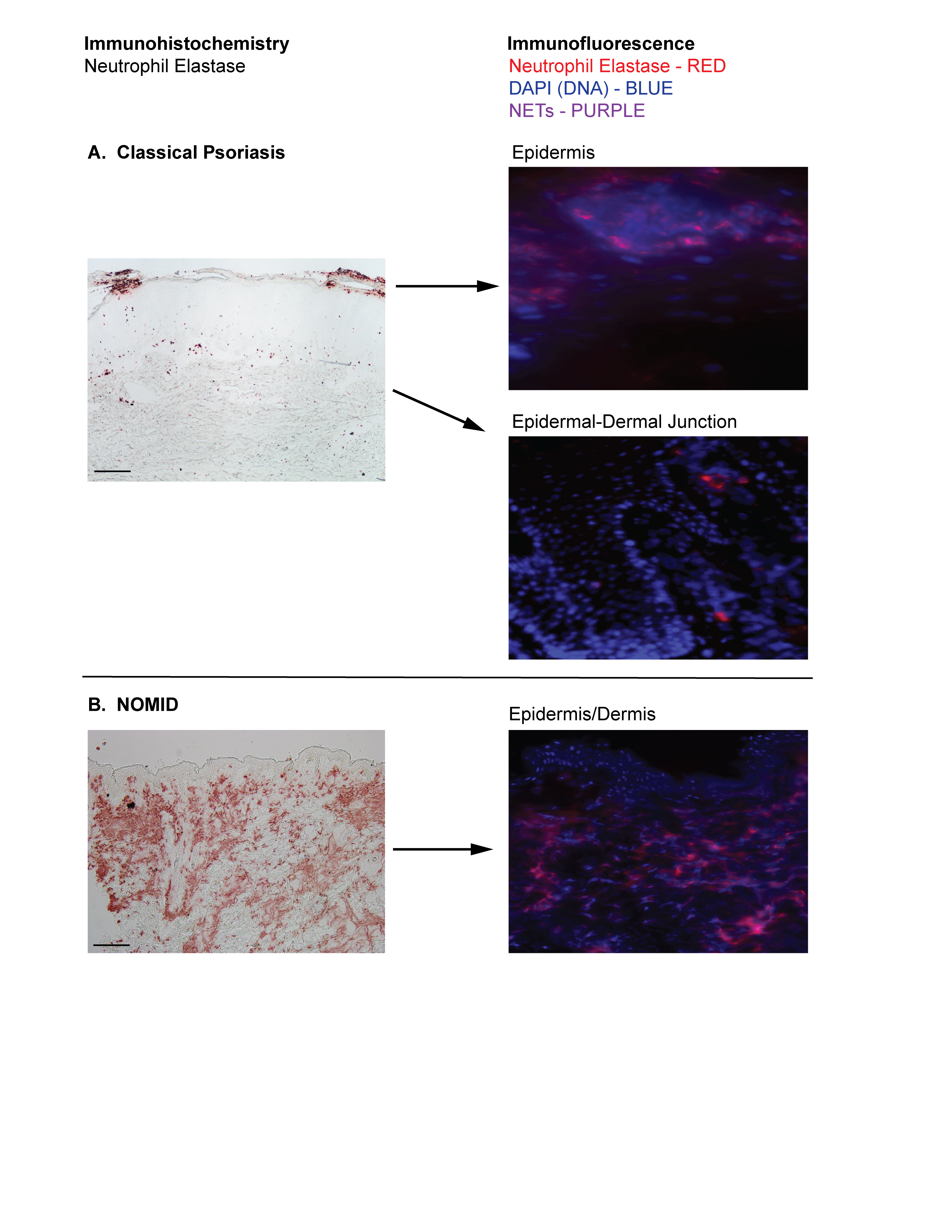

Supplement: Figure S2 — Neutrophil Extracellular Trap (NET)-like structures are found in NOMID. Frozen sections from patients with A. psoriasis, or B. NOMID were used to stain for neutrophil elastase via immunohistochemistry (left), as well as immunofluorescence of neutrophil elastase (red) and DNA (DAPI) (blue; right, n = 3 for NOMID). NET-like structures can be seen as purple co-localization of neutrophil elastase and DNA. A. In psoriasis, these NET-like structures were seen predominantly in the epidermis. B. In NOMID, NET-like structures were seen within the dermis. All images are shown at 20×, size bar is 100 µm. (TIF) [file pone.0049408.s002.tif]

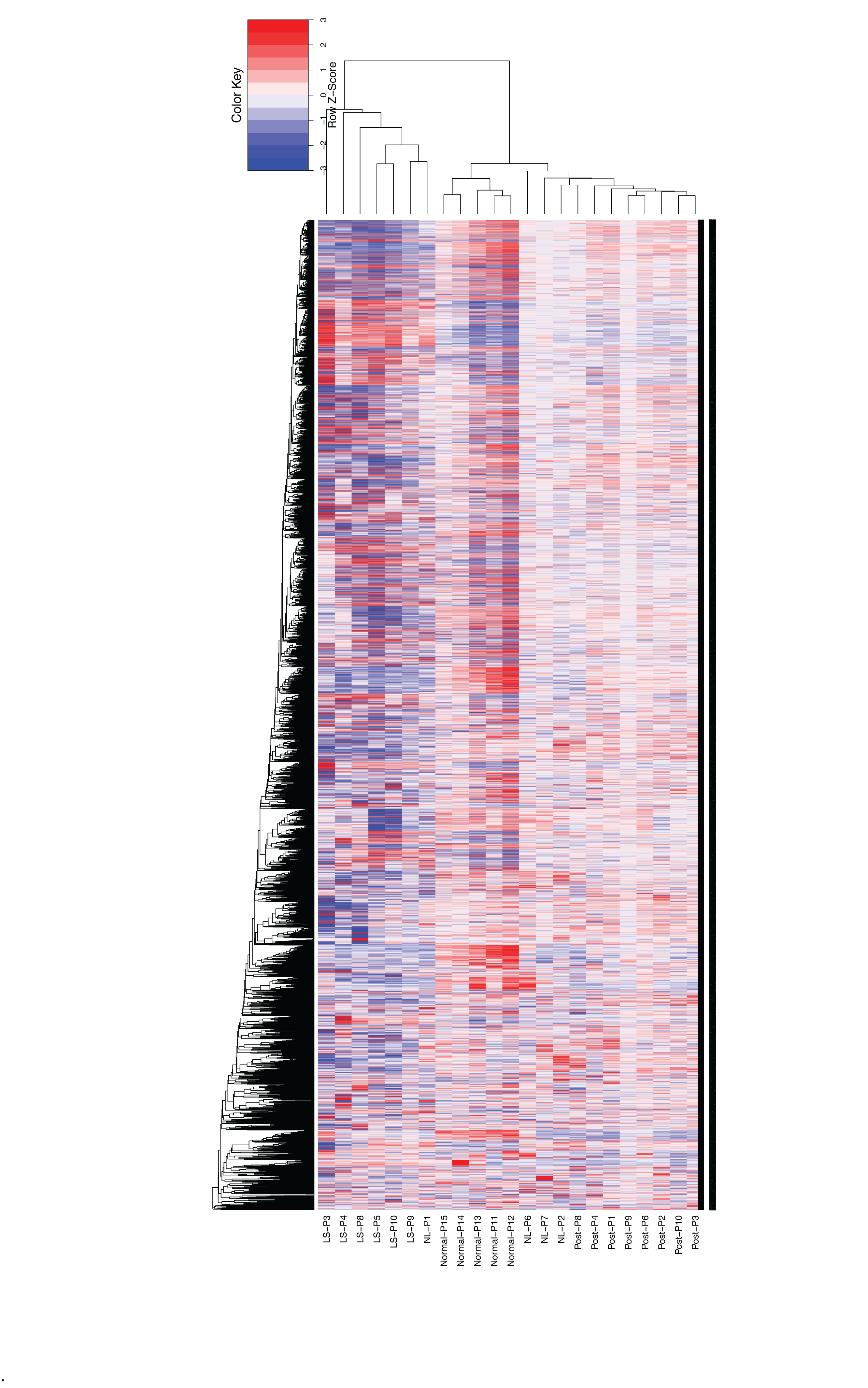

Supplement: Figure S3 — Heat map for DEGs in study. Clustering of patients into lesional (LS), pre-treatment non-lesional (NL in figure, pre-NL in text), normal, and post-treatment non-lesional (post, post-NL) groups. (TIF) [file pone.0049408.s003.tif]
