# Supplementary material for: Homeostatic Tissue Responses in Skin Biopsies from NOMID Patients with Constitutive Overproduction of IL-1β
Source: PLoS One. 2012 Nov 30;7(11):e49408. doi: 10.1371/journal.pone.0049408 (PMC3511496; doi:10.1371/journal.pone.0049408)
Supplement: Methods S1 — (DOC) [file pone.0049408.s009.doc]

**Supplementary Material and Methods (SMM)**

***Skin samples for study.*** Skin biopsies were cut in two: half was stored in OCT for cryosections, and half snap-frozen in liquid nitrogen for RNA extraction. Some LS biopsies were processed for paraffin sections. Neonatal normal foreskins were used as normal controls for immunohistochemistry studies. Archived normal skin RNA samples collected at Rockefeller University were used as controls in the genomic studies. The classical urticaria-like rash of NOMID was present in all patients with pre-treatment LS biopsies. While all patients had rashes before treatment was initiated, only one baseline biopsy (LS or pre-NL) was collected in order to minimize distress for the young patients. Pre-NL biopsies were taken from clinically unaffected areas of skin, and post-treatment biopsies were also NL (post-NL)**.** Patient demographics and skin phenotype and steroid and Anakinra dose at the time of biopsy are listed in Table S5. Asterisks marks biopsies included in the microarray analyses.

Limitations to our study include the inability to validate IPA pathways by RT-PCR expression data, and the variable amount of steroids patients were on at the time of biopsy, with higher doses pre-treatment than post-anakinra treatment. The research biopsies we were able to obtain from children were small and left us with insufficient sample to conduct confirmation studies. However, the dynamic and gradual change in the three lesional tissue states (LS, pre-NL and post-NL) internally validates our expression data. As steroids could modify gene expression profiles, we statistically corrected for an effect on downregulated genes by conducting our analysis both with and without steroid dose as a co-variable without a change in the pathways.

***Immunohistochemistry and Immunofluorescence.*** Tissue sections were stained with haematoxylin and eosin (n=14) in a standard manner. Leukocyte cell marker staining was performed on frozen sections, and positive cells counted as previously published . A list of antibodies is in Table S6. Paraffin sections were stained after antigen retrieval using DIVA Decloaker (Biocare, Concord, CA). For two color immunoflouresence, paraffin LS skin sections in patients with *CIAS1* mutations were stained using antibodies to cleaved caspase-1, and to CD11c and CD163 (Table S6) (n=3). A Hoechst stain was used to counterstain nuclei. Widefield immunofluorescence images were acquired using appropriate filters on a Leica DMR microscope with a Hamamatsu C4742-95 digital camera (Bridgewater, NJ) controlled by IVision software. Hematoxylin and Eosin (H&E) of normal skin is an archived image for comparison (Fig. S1A). Negative control is minus primary. IL-1Ra (Fig. S1B) and IL-36Ra (Fig. S1C) immunohistochemistry were performed on a subset of normal skin and NOMID frozen sections (n=3). For neutrophil extracellular trap (NET) immunofluorescence staining, parallel frozen sections of lesional psoriasis (n=1) and NOMID (n=3) were co-stained with neutrophil elastase and DAPI (Vector Laboratories, Burlingame, CA).

***RNA preparation and Gene Array.*** It was anticipated that the yield of RNA from these small biopsies would be low, so RNA was extracted from NOMID skin samples using the RNeasy Micro Kit (Qiagen, Valencia, CA). For the normal healthy controls, the RNeasy Mini kit (Qiagen) was used. NuGen system (San Carlos, CA) with Affymetrix Human Gene 1.0 ST arrays (Santa Clara, CA) were chosen to amplify RNA for their superior performance with suboptimal RNA quality. This method uses a random primer system for amplification of RNA. Chips were scanned in the GS3000 scanner (Affymetrix). Five normal, 4 NL, 6 LS, and 8 post treatment samples were processed for microarray analysis. There was insufficient RNA available for RT-PCR studies.

***Statistical Analysis.*** The statistical analysis was carried out in the R language version 2.12 ([www.r-project.org](http://www.r-project.org/)), and packages were from the Bioconductor project ([www.bioconductor.org](http://www.bioconductor.org/)). The pathway analysis was performed in the ingenuity pathway analysis ([www.ingenuity.com](http://www.ingenuity.com/)) tool, and cell count analysis in Graphpad Prism (GraphPad Software, California). For gene array analysis, RMA algorithm and *xps* package from Bioconductor was used for preprocessing. After background correction, instead of the default quantile normalization, we used robust quantiles normalization. The reference distribution (to which all chips are normalized) calculated by quantiles normalization has very narrow distribution (small SD), due mainly to 3 chips that had narrow intensity distribution. By using robust quantiles normalization, the reference distribution to which all chips are normalized, is calculated not considering outlier chips. This rendered a normalization in the normal range for this chip series. Probesets with SD> 0.4 and at least one sample with expression >3 were included in the analysis. To identify differentially expressed genes we used the *limma package*. A mixed effect model with fixed effect Group and Covariate Steroids and random intersect for Patient was used. This corrected for steroid dose and assessed the one patient who was not on steroids pretreatment. A major effect of steroids could not be seen. A linear model with fixed factor Group and random intersect for Patient and steroid dose was used to model the data.

Genes differentially expressed in the comparisons of interest were identified using a moderated two-tailed t-test. The resultant *P*-value was adjusted for multiple hypotheses testing, controlling the false-discovery rate (FDR) using the Benjamini-Hochberg procedure. Genes with FDR<0.05 and FCH>2 were considered differentially expressed. Differentially expressed genes (DEGs) were queried for over-represented canonical pathways using Ingenuity Pathway analysis and biological networks. Several IL-1 family members or signaling molecules did not pass the filter and therefore did not have measurable gene expression. This is not uncommon in low expressing genes such as cytokines .

For immunohistochemistry data, a Student’s t-test was used to compare immunohistochemistry cell counts in the four groups, and significance was accepted as p<0.05.

***Description of Supplementary Tables***

**Table S1** gives the list of differentially expressed genes (DEGs) for each comparison, for Fold Change greater than 2 (FCH>2), and False Discovery Rate (FDR) less than 0.05.

**Table S2** includes the average level of gene expression, or “mean per group” (log2) for each tissue state.

**Table S3 Ingenuity Pathways Analysis (IPA).**

**Table S3A** summarizes the over-represented canonical Ingenuity Analysis pathways identified by the upregulated DEG in the LS versus Normal comparison. The number of DEGs in the pathway are indicated, and the percent of total genes in the pathway in brackets. DEGs for this and other comparisons were then reviewed, and up- and down-regulated DEGs identified in those pathways are listed. *Italics* indicates that the pathway was significant.

**Table S3B** summarizes the over-represented canonical Ingenuity Analysis pathways in LS versus pre-NL comparison, and then their presence in the other comparisons is shown. The number (and percentage) of upregulated (red column) or downregulated (green column) genes in each pathway for each comparison are listed.

**Table S4** is the residual subclinical disease profile, defined as percent improvement in DEGs (LS versus Normal) week 12 , with improvement below 75% considered meaningful (identified in Table S6 as pink boxes). This is a slightly different calculation than in the psoriasis case. In Suárez-Fariñas, *et al*. , the genomic resolution of psoriasis after treatment (termed the “residual disease genomic profile” or RDGP) was evaluated by defining the improvement after treatment of the psoriasis phenotype defined as the DEG between LS and NL skin . In the present NOMID study we defined the disease genomically as the DEG between LS vs Normal. To measure improvement with treatment with the IL-1 blocking agent anakinra, we defined improvement in this set of genes as the ratio between Post vs LS (Treatment Effect) and LS vs Normal (disease phenotype), ie: . $100\times\left(sign\left(X_{LS}-X_{Normal}\right)\right)\frac{X_{Post}-X_{LS}}{X_{LS}-X_{Normal}}$ , were X_Post_, X_LS_, X_Normal_ represents the expression values (in log2-scale) of a gene at POST, LS and Normal respectively. Genes with an improvement of less than 75% were considered as the residual subclinical disease profile.

The number of unique genes in this residual subclinical disease profile in NOMID is greater than DEGs in the post-NL versus Normal comparison (Table S3) due to the nature of the calculations. For example, a gene that has a level of expression of 2 at LS compared to 0 at N, that is reduced to 0.9 post-NL, would appear in the DEGs for Lesional versus Normal (2FCH), but not in the post-NL versus Normal, and it would be improved by 1.1/2 (55%) and would thus be part of the residual subclinical disease profile.

**Table S5.** Clinical details of patients included in the study.

**Table S6.** Antibodies used in this study.

**References**

1. Fuentes-Duculan J, Suárez-Fariñas M, Zaba LC, Nograles KE, Pierson KC, et al. (2010) A subpopulation of CD163-positive macrophages is classically activated in psoriasis. J Invest Dermatol 130: 2412-2422.

2. Suárez-Fariñas M, Lowes MA, Zaba LC, Krueger JG (2010) Evaluation of the psoriasis transcriptome across different studies by gene set enrichment analysis (GSEA). PLoS One 5: e10247.

3. Suárez-Fariñas M, Fuentes-Duculan J, Lowes MA, Krueger JG (2010) Resolved psoriasis lesions retain expression of a subset of disease-related genes. J Invest Dermatol 131: 391-400.
