# Supplementary material for: Homeostatic Tissue Responses in Skin Biopsies from NOMID Patients with Constitutive Overproduction of IL-1β
Source: PLoS One. 2012 Nov 30;7(11):e49408. doi: 10.1371/journal.pone.0049408 (PMC3511496; doi:10.1371/journal.pone.0049408)
Supplement: Table S3 — Ingenuity Pathways Analysis (IPA). (PDF) [file pone.0049408.s012.pdf]

Ingenuity Pathways Analysis (IPA): Canonical pathways

A. INITIAL ANALYSIS BY DEGs in LS versus Normal "UP"			LS vs Normal UP	LS vs Normal DOWN	pre-NL vs Normal UP	PRE-NL vs Normal DOW	post-NL vs Normal UP	post-NL vs Normal DOW	TOTAL # GENES IN PATHWAY
Acute Phase Response Signaling			***13 (8)***	***44 (27)***	1 (0.6)	18 (11)		6 (4)	164
Amyloid Processing			***6 (11)***	16 (29)		6 (11)			56
IL-17 Signaling			***7 (9)***	***22 (30)***		8 (11)			74
IL-6 Signaling			***8 (9)***	***25 (28)***		9 (10)		3 (3)	90
Ovarian Cancer Signaling			***11 (11)***	31 (30)		9 (9)			102
Role of NANOG in Mammalian Embryonic Stem Cell Pluripotency			***11 (11)***	27 (27)		***12 (12)***			100
Role of Osteoblasts, Osteoclasts and Chondrocytes in Rheumatoid Arthritis			***18 (10)***	48 (26)		***22 (12)***		6 (3)	188
Wnt/β-catenin Signaling			***15 (11)***	39 (28)		***11 (8)***		5 (4)	140
Role of Macrophages, Fibroblasts and Endothelial Cells in Rheumatoid Arthritis			***24 (14)***	63 (37)	1 (0.6)	29 (17)			171

B. INITIAL ANALYSIS BY DEGs LS vs pre-NL "DOWN"	LS vs pre-NL DOWN	LS vs pre-NL UP	LS vs Normal UP	LS vs Normal DOWN	pre-NL vs Normal UP	PRE-NLvN DOWN	post-NL vs Normal UP	post-NL vs Normal DOW	TOTAL # GENES IN PATHWAY
Cleavage and Polyadenylation of Pre-mRNA	***5 (42)***			***9 (75)***		2 (17)			12
Mechanisms of Viral Exit from Host Cells	***13 (29.5)***		4 (9)	***16 (36)***		5 (11)			44
EGF Signaling	***14 (29)***			***21 (43)***		***8 (16)***			49
Oxidative Phosphorylation	***44 (26.7)***			***69 (42)***	1 (0.6)			9 (5)	166
DNA Methylation and Transcriptional Repression Signaling	***6 (26)***			***11 (48)***					23
NRF2-mediated Oxidative Stress Response	***44 (24)***			***73 (40)***		***23 (13)***			183
Protein Ubiquitination Pathway	***48 (24)***			***92 (46)***		***33 (16)***		13 (7)	201
Mitochondrial Dysfunction	***41 (23.7)***			***59 (35)***					171
Clathrin-mediated Endocytosis Signaling	***39 (23.4)***			***58 (35)***		***20 (12)***			167
CNTF Signaling	***12 (23)***			***19 (37)***		7 (14)			52
VEGF Signaling	***22 (23)***			***36 (37)***		12 (12)			97
EIF2 Signaling	***22 (22)***			***44 (44)***		***16 (16)***			100
FAK Signaling	***22 (22)***			***33 (34)***		10 (10)			98
RAN Signaling	***5 (22)***			***10 (44)***		3 (13)			23
IL-9 Signaling	***8 (22)***		2 (5)	9 (24)	1 (3)	3 (8)			37
IGF-1 Signaling	***21 (21)***		5 (5)	***35 (35)***		***15 (15)***			100
GM-CSF Signaling	***14 (21)***	1 (1.5)	4 (6)	***24 (36)***		9 (13)			67
Docosahexaenoic Acid (DHA) Signaling	***10 (21)***	1 (2)		***15 (31)***		6 (12.5)			48
IL-3 Signaling	***15 (21)***		4 (6)	***23 (32)***	1(1)	9 (12.5)			72
IL-2 Signaling	***12 (21)***		3 (5)	***20 (35)***		8 (14)			58
JAK/Stat Signaling	***13 (20)***			***22 (34)***		8 (12.5)			64
Virus Entry via Endocytic Pathways	***19 (20)***			***31 (32)***		12 (12.5)			96
PDGF Signaling	***15 (20)***			***27 (36)***		***11 (15)***			76
TR/RXR Activation	***19 (20)***			***27 (28)***	2 (2)	11 (11)	1 (1)		97
