# Supplementary material for: Homeostatic Tissue Responses in Skin Biopsies from NOMID Patients with Constitutive Overproduction of IL-1β
Source: PLoS One. 2012 Nov 30;7(11):e49408. doi: 10.1371/journal.pone.0049408 (PMC3511496; doi:10.1371/journal.pone.0049408)
Supplement: Table S5 — Clinical details of patients included in the study. (PDF) [file pone.0049408.s014.pdf]

Clinical details

	Age	Sex	Type	Clinical Phenotype pre-treatment	Bx site	Mutation	Other NOMID features	Anakinra dose at time of post treatment biopsy	Prednisone equivalent steroid dose (mg/kg) pre/post
	11yo*	M	lesional/post	Generalized, scattered urticarial slightly pink eruption from head to toe.	R buttock/R hip	negative	bony overgrowth, hearing and vision loss, aseptic meningitis, developmental delay	Anakinra 2mg/kg/dy	0.46/0.31
	9yo*	F	lesional/post	Uritcarial plaques scattered on head, trunk, extremities, buttocks, 10-15% TBSA.	buttock/ R upper buttock	D303N	aseptic meningitis	Anakinra 1.5mg/kg/dy	0.29/0.20
	8yo*	F	lesional/post	face, chest, arms, back, legs, buttocks with numerous small (8mm to 1.5 cm) annular urticarial lesions	L buttock/ R buttock	G569R	bony overgrowth, aseptic meningitis	Anakinra 1.5mg/kg/dy	0.55/0.30
	4yo*	M	lesional/post	Diffuse pink-brown urticarial rash that is generalized from head to toe.	R upper back/ L buttock	G326E	hearing loss, aseptic meningitis	Anakinra 1.5mg/kg/dy	0/0
	7yo	M	lesional/post	Pink, scattered, somewhat (2mm-1cm) reticulated (or lacy) wheals on the upper chest, back, thigh, palms and soles.	R buttock/R upper buttock	negative	bony overgrowth, aseptic meningitis	Anakinra 1.5mg/kg/dy	0.44/0.38
	7yo*	F	lesional	Scattered urticarial papules varying in size from 1 mm to more elongated lesions with a long-diameter of 1.5 cm. Erythema cheeks.	Unknown	L264F	bony overgrowth, hearing loss, aseptic meningits, mild developmental delay	N/A	1.04
	8yo*	F	nonlesional	Generalized reticulation on the upper trunk, knees and back. No evidence of NOMID like rash, nails have clubbed appearance.	R buttock	A374N	hearing loss, aseptic meningitis, severe developmental delay	N/A	0.83
	16yo	M	lesional	few discrete pink, urticarial papules varying from 6-10mm over the lower abdomen and over the gluteal area.	Abdomen left lower quadrant	F443L	hearing loss, aseptic meningitis, mild developmental delay	N/A	0.16
	4yo*	F	nonlesional/post	Scattered erythematous macules in groups. LE thighs, flanks <1% BSA	R upper buttock	negative	bony overgrowth, visual impairment, developmental delay	Anakinra 2mg/kg/dy	0/0
	16yo*	M	nonlesional/post	Generalized non-pruritic urticaria rash.	L arm/unknown	T348M	hearing loss, aseptic meningitis	Anakinra 1.5mg/kg/dy	0/0
	28yo	F	nonlesional/post	Chest, face, arms, back, abd, legs with few pink urticarial papulaes.	L upper inner arm/unknown	D303N	hearing loss, visual impairment, aseptic meningitis, mild developmental delay	Anakinra 1.5mg/kg/dy	0/0
	18yo	F	post	Erythema noted on eyelids.No rash noted today. No urticaria.	Unknown	negative	bony overgrowth, hearing loss, aseptic meningitis, developmental delay	Anakinra 2mg/kg/dy	0.08
	8yo*	M	lesional/post	Irregularly shaped pink urticarial papules varying from a few mm's to 1 cm in diameter scattered on his extremities. Palms with mottled bright erythematous macules.	R buttock/R iliac crest	L632F	hearing loss, aseptic meningitis	Anakinra 2mg/kg/dy	0.97/0.67
	11yo*	F	nonlesional/post	keratosis pilaris, lichenification antecubital fossae, Xerosis	R buttock/R buttock	V262A	hearing loss, aseptic meningitis, mild developmental delay	Anakinra 1.5mg/kg/dy	0/0

*biopsies included in the microarray analyses
