# Supplementary material for: Homeostatic Tissue Responses in Skin Biopsies from NOMID Patients with Constitutive Overproduction of IL-1β
Source: PLoS One. 2012 Nov 30;7(11):e49408. doi: 10.1371/journal.pone.0049408 (PMC3511496; doi:10.1371/journal.pone.0049408)
Supplement: Table S6 — Antibodies used in this study. (PDF) [file pone.0049408.s015.pdf]

## Table S6. Antibodies used for Immunohistochemistry and Immunofluorescence.

| Antibody | Cell specificity | Source | Clone[a] | Iso | Dilution |
|---|---|---|---|---|---|
| CD11c | myeloid DC (dermal) | BD Pharmingen | B-ly6 | IgG1 | 1:100 |
| BDCA-1 (CD1c) | myeloid DC (dermal/resident) | Miltenyi | AD5-8E7 | IgG2a | 1:100 |
| HLA-DR | activated lymphocytes, monocytes, macrophages and dendritic cells | BD Pharmingen | L243 | IgG2a | 1:200 |
| CD163 | macrophages | Acris | 5C6-FAT | IgG1 | 1:500 |
| CD3 | T lymphocytes | BD Pharmingen | SK7 | IgG1 | 1:100 |
| NALP3 | myeloid DC, keratinocytes, macrophages, monocytes | Acris | Nalpy3-b | IgG1 | 1:50 |
| iNOS | variety of cell types | Santa Cruz Biotechnology | N-20 | Affinity purified rabbit | 1:20 |
| IL-23p19 | myeloid DC, monocytes, macrophages | BioLegend | HLT2736 | IgG1 | 1:50 |
| Neutrophil elastase | Neutrophils | Dako | NP57 | IgG1 | 1:200 |
| Cleaved caspase-1 p20 (h297) | Activated caspase-1 | Santa Cruz Biotechnology | Poly-clonal goat IgG | N/A | 1:20 |
| IL-1Ra | Interleukin 1 receptor antagonist | R&D Systems | Poly-clonal goat IgG | N/A | 1:200 |
| IL-36Ra (IL-1F5) | Interleukin 36 receptor antagonist (IL-1F5) | R&D Systems | 190524 | IgG2b | 1:10 |

[a]All are murine monoclonals unless stated
